# Experimental and Modeling Study on Methane Hydrate Equilibrium Conditions in the Presence of Inorganic Salts

**DOI:** 10.3390/molecules29153702

**Published:** 2024-08-05

**Authors:** Qiang Fu, Mingqiang Chen, Weixin Pang, Zhen Xu, Zengqi Liu, Huiyun Wen, Xin Lei

**Affiliations:** 1 State Key Laboratory of Offshore Natural Gas Hydrate, China National Offshore Oil Corporation, Beijing 100028, China; fuqiang8@cnooc.com.cn (Q.F.); pangwx@cnooc.com.cn (W.P.); wenhy@cnooc.com.cn (H.W.); leixin4@cnooc.com.cn (X.L.); 2 Research Institute of China National Offshore Oil Cooperation, China National Offshore Oil Corporation, Beijing 100028, China; 3 State Key Laboratory of Heavy Oil Processing, China University of Petroleum-Beijing at Karamay, Karamay 834000, China; liuzq@cupk.edu.cn

**Keywords:** inorganic salts, methane hydrate, inhibition, thermodynamics model

## Abstract

The aim of this study was to determine the influence of four inorganic salts, KCl, NaCl, KBr and NaBr, on the thermodynamic conditions of methane hydrate formation. In order to achieve this, the vapor–liquid water-hydrate (VL_W_H) equilibrium conditions of methane (CH_4_) hydrate were measured in the temperature range of 274.15 K–282.15 K by the isothermal pressure search method. The results demonstrated that, in comparison with deionized water, the four inorganic salts exhibited a significant thermodynamic inhibition on CH_4_ hydrate. Furthermore, the inhibitory effect of Na^+^ on methane hydrate is more pronounced than that of K^+^, where there is no discernible difference between Cl^−^ and Br^−^. The dissociation enthalpy ($∆H_{diss}$) of CH_4_ hydrate in the four inorganic salt solutions is comparable to that of deionized water, indicating that the inorganic salt does not participate in the formation of hydrate crystals. The Chen–Guo hydrate model and N–NRTL–NRF activity model were employed to forecast the equilibrium conditions of CH_4_ hydrate in electrolyte solution. The absolute relative deviation (AARD) between the predicted and experimental values were 1.24%, 1.08%, 1.18% and 1.21%, respectively. The model demonstrated satisfactory universality and accuracy. This study presents a novel approach to elucidating the mechanism and model prediction of inorganic salt inhibition of hydrate.

## 1. Introduction

Natural gas hydrate is a non-stoichiometric crystalline compound formed by small gas molecules, like methane (guest molecules) and water molecules (host molecules), under high pressure and low temperature [1,2]. The water molecules create cavities in the crystal structure by hydrogen bonding, while the guest molecules are trapped in the cavities in the crystal structure [3]. The crystal structure of natural gas hydrates consists mainly of structure I, structure II and structure H, which is determined by the diameter of the guest molecules [4]. Hydrate poses significant challenges to the oil and gas industry. The hydrates can accumulate in pipelines and equipment, leading to flow blockages and operational disruptions. Effective inhibition strategies are therefore essential to mitigate hydrate formation and ensure safe hydrocarbon production and transport [5,6].

The inhibition of natural gas hydrates primarily involves two main approaches: thermodynamic inhibition and kinetic inhibition [7]. Thermodynamic inhibitors inhibit the hydrate formation by changing the phase equilibrium conditions, such as reducing the temperature required for hydrate formation or increasing the pressure required for hydrate formation, but their disadvantages are large dosage and high cost [8]. These inhibitors include salts, alcohols, etc. [9,10,11]. Li et al. [12] studied the phase equilibrium conditions of methane hydrate in chloride salt system, and the results showed that MgCl_2_ inhibition was stronger than that of other salts. Semenov et al. [13,14] studied the influence of different concentrations of methanol on methane hydrate and determined that methanol has thermodynamic inhibition of methane hydrate in a specific concentration range. Kinetic inhibitors work by affecting the kinetics of hydrate formation rather than the phase equilibrium [5,11]. Zhang et al. [15] observed the growth kinetics of methane hydrate by using a confocal Raman imaging microscope and found that lactam-based PVCap effectively delayed the growth of hydrate from the gas–liquid boundary to the bulk solution direction, while non-lactam PNIPAM works better on prolonging the time for water molecules to develop a well-ordered hydrate structure. Farhadian et al. [5] showed that kinetic inhibitors with piperazine groups have good hydrate inhibition, even better than poly (N-vinyl caprolactam) in acidic solutions. These inhibitors interfere with crystal growth, inhibit nucleation, or modify the hydrate crystal surface to prevent agglomeration and adherence to pipeline walls. Examples of kinetic inhibitors include polymers, surfactants, and certain organic compounds that adsorb to the hydrate surface and disrupt the hydrate crystal lattice structure [16]. At present, thermodynamic inhibition is still a reliable method in industrial applications, and there is a need for further research under different application scenarios.

Recent research advances in natural gas hydrate inhibition have focused on several key areas to enhance inhibition efficiency and reduce environmental impact [17,18]. One significant area of advancement is the development of hybrid inhibitors that combine both thermodynamic and kinetic inhibition mechanisms. These hybrid inhibitors exploit the strengths of each approach to provide more robust protection against hydrate formation over a wider range of operating conditions. By synergistically lowering the thermodynamic hydrate equilibrium and inhibiting kinetic growth, these compounds offer improved performance and reduced inhibitor dosage requirements [19]. Furthermore, researchers have explored novel chemical formulations and composite materials to enhance hydrate inhibition effectiveness. Advances in molecular design and synthesis have led to the discovery of new inhibitor compounds with superior performance characteristics, such as increased stability, lower toxicity, and enhanced selectivity for hydrate inhibition [20]. Computational methods, including molecular modelling and simulation techniques, have played a key role in guiding the design and optimization of these inhibitors by providing insights into molecular interactions and inhibitor efficacy at the atomic level [21].

In addition, advancements in understanding the mechanisms of hydrate formation and inhibitor performance have been facilitated by experimental studies conducted in high-pressure laboratories and field trials [22]. These studies provide essential data on inhibitor efficiency, allowing researchers to validate theoretical models and refine inhibitor formulations accordingly. Field trials in offshore and subsea environments have demonstrated the practical applicability and operational reliability of new inhibitor technologies, paving the way for their commercial deployment in the oil and gas industry [23,24].

As the industry moves towards deeper offshore and remote operations, the demand for reliable hydrate inhibition methods will only intensify. NaCl is the main inorganic salt in seawater, so it is necessary to explore the effect of NaCl on inhibiting methane hydrate for natural gas pipeline transportation in the process of hydrate exploitation in sea areas. At the same time, KCl, NaBr and KBr were selected as the control group in order to explore the mechanism of inorganic salt inhibiting hydrate and to clarify the difference between cation and anion inhibiting hydrate. The seafloor temperature of the hydrate deposit area in the South China Sea is about 275.15 K, and the water depth is about 1200 m. According to the water temperature gradient, the temperature of the sea decreases by 0.6 K every 100 m, so the temperature range from sea level to the seafloor is 275.15 K to 280.35 K. The study presents new experimental data on the equilibrium of VL_W_H on CH_4_ hydrate in the presence of four inorganic salts (KCl, NaCl, KBr, NaBr) at a range of temperatures (274.15 K–282.15 K). In order to assess the reliability of the VLWH equilibria data, a thermodynamic consistency assessment was conducted. The thermodynamic inhibition of four inorganic salts on CH_4_ hydrate was evaluated in detail. The change in enthalpy of dissolution ($∆H_{diss}$) was calculated using the Clausius–Clapeyron relation in order to provide a quantitative description of the effect of inorganic salts on methane hydrate crystals. A prediction model of VL_W_H equilibrium conditions was established on the basis of the Chen–Guo model and the N–NRTL–NRF activity model. By analyzing the VL_W_H equilibrium conditions of four types of inorganic salts, cations are the main way to inhibit hydrate. This provides a direction for the subsequent synthesis of highly effective inhibitors. At the same time, a high precision thermodynamic prediction model is established, which lays a foundation for practical application.

## 2. Results and Discussion

### 2.1. The VL_W_H Equilibrium Conditions in the Presence of Inorganic Salts

**Figure 1:**
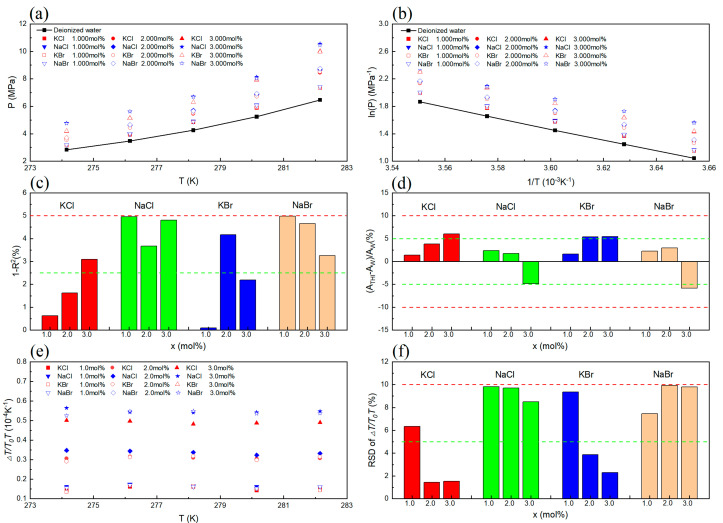
Thermodynamic consistency of CH_4_ hydrate formation conditions in the presence of inorganic salts. (**a**) VL_W_H of CH_4_ hydrate in different inorganic salt solutions. (**b**) Linear relationship between ln(*P*) and 1/*T* in different inorganic salt solutions. (**c**) The result of linear consistency assessment. (**d**) The result of $∆H_{diss}$ consistency assessment. (**e**) The values of $\frac{∆T}{T_{0}T}$ in different inorganic salt solutions. (**f**) The result of $a_{w}$ consistency assessment.

The VL_W_H equilibrium conditions of CH_4_ in inorganic salt systems have been delineated in detail within Table 1. The validation of the experimental data’s thermodynamic consistency was meticulously conducted through the methodology advocated by Sa et al. [25]. The detailed derivation of the models is shown in Appendix A. Three distinct categories of evaluation outcomes manifesting this validation process have been visually represented in Figure 1.

Figure 1a reveals that the presence of KCl, NaCl, KBr, and NaBr results in elevated phase equilibrium pressure values for CH_4_ hydrate. This evidence suggests a thermodynamic inhibitory effect of KCl, NaCl, KBr, and NaBr on CH_4_ hydrate. Figure 1b depicts the linear correlation between 1/*T* and ln(*P*), and Figure 1c illustrates the fit of the experimental data points to the linear regression equation. The values of 1 − *R*^2^, which represent the degree of linear inconsistency, were found to be below 5%. This suggests that the experimental data exhibited satisfactory consistency. As can be seen from the results shown in Figure 1d, some of the values of ($A_{THI}$ − $A_{W}$)/$A_{W}$ are higher than 5% but lower than 10%, which is an indication that the experimental data passed the evaluation of dissociation enthalpy consistency. The values of $\frac{∆T}{T_{0}T}$ for different inorganic salt types and concentration conditions are shown in Figure 1e. The RSD of $\frac{∆T}{T_{0}T}$ was also calculated. The results are shown in Figure 1f. From the results, it can be seen that some of the RSD values are higher than 5% but lower than 10%, which is an indication that the experimental data passed the evaluation of water activity consistency.

Notably, the VL_W_H equilibrium data successfully conform to the criteria stipulated by the three thermodynamic consistency assessments. This achievement not only underscores the robustness of the data but also eschews any discrepancies, implying their credibility for subsequent analyses pertaining to hydrate inhibition effects and the formulation of phase equilibrium models.

### 2.2. Thermodynamic Inhibition of Inorganic Salts on CH_4_ Hydrate

**Figure 2:**
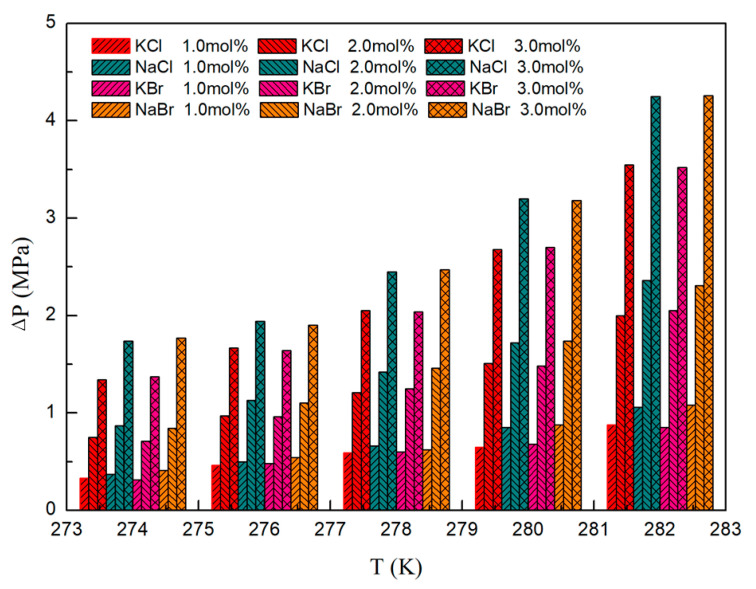
∆P values of CH_4_ hydrate in the presence of inorganic salts at different conditions.

The investigation encompassed an analysis of the impact exerted by KCl, NaCl, KBr, and NaBr on the VL_W_H equilibrium pressure of CH_4_ hydrate across the temperature span of 274.15 K to 282.15 K. The parameter ∆P, signifying the disparity between the phase equilibrium pressure of CH_4_ hydrate within the inorganic salt system and that within the deionized water system, was meticulously examined, as illustrated in Figure 2.

Relative to the deionized water system, the VL_W_H equilibrium pressure of CH_4_ hydrate within 1.00 mol%, 2.00 mol%, and 3.00 mol% KCl solutions exhibited elevations of 12.94%, 28.49%, and 49.88%, respectively. Correspondingly, in NaCl solutions, these increments were observed to be 14.79%, 32.40%, and 61.19%, sequentially. Similarly, in KBr solutions, the increments were noted to be 12.98%, 28.38%, and 49.85%, respectively. In NaBr solutions, the enhancements were recorded at 14.76%, 32.93%, and 60.20%, respectively. These findings underscore the pronounced thermodynamic inhibitory effects of KCl, NaCl, KBr, and NaBr on CH_4_ hydrate formation. At the same time, according to the data results, it can be found that, with the increase of temperature, the ∆P also increases. This change law means that the intensity of inorganic salt inhibition on hydrate is positively correlated with temperature. The observed phenomena are posited to be attributed to the interaction of electrolytes with water molecules, forming solvated shells of ions, consequently diminishing water activity and impeding hydrate formation.

Upon juxtaposing the KCl and NaCl systems, discernible trends unveil a more potent inhibition effect of NaCl on methane hydrate in contrast to KCl. This disparity in inhibition effect also persists in the comparison of KBr and NaBr systems, implying a stronger thermodynamic inhibition effect exerted by Na^+^ ions relative to K^+^. Comparing KCl and KBr systems, it is found that KCl has the same inhibition effect on methane hydrate as KBr systems, and this result also exists in the comparison between NaCl and NaBr systems. This indicates that the thermodynamic inhibition effect of Cl^−^ on methane hydrate is comparable to that of Br^−^.

### 2.3. The Hydrate $∆H_{diss}$ in the Presence of Inorganic Salts

The $∆H_{diss}$ can be effectively elucidated through the Clausius–Clapeyron equation in the presence of inhibitors. Table 2 presents a succinct comparison of $∆H_{diss}$ for CH_4_ hydrate within the realms of deionized water, KCl, NaCl, KBr, and NaBr solutions. It is noteworthy that the calculated $∆H_{diss}$ within deionized water, derived from the Clausius-Clapeyron equation, is −59.42 kJ·mol^−1^. This value is situated between the −56.9 kJ·mol^−1^ reported by Sloan et al. and the −62.85 kJ·mol^−1^ documented by Skovborg et al. These results, which have been obtained through computational means, are now ready for further comprehensive analysis.

The outcomes demonstrate that the computed $∆H_{diss}$ of CH_4_ hydrate within the four inorganic salt solutions yields values that are similar to those observed in deionized water, with relative deviations of 2.33%, 1.46%, 0.16%, and 1.42%, respectively. It is noteworthy that, for thermodynamic hydrate inhibitors (THIs) that have no impact on the crystal structure of the hydrate, the $∆H_{diss}$ values remain constant regardless of variations in the type and concentration of THIs. This strongly suggests that the inorganic salt molecules do not participate in the actual formation of hydrate crystal structures but rather are exclusively present within the liquid aqueous phase. With the formation and growth of hydrate crystals, a large number of water molecules are consumed. The analysis of dissociation enthalpy shows that inorganic salts do not participate in the formation of hydrate crystals and thus accumulate in the liquid phase. The higher the concentration of inorganic salts in the liquid phase, the lower the activity of water. Therefore, the formation conditions of hydrate will be more stringent, and the crystallization of hydrate will be further inhibited.

### 2.4. Prediction of the VL_W_H Equilibrium Conditions

**Figure 3:**
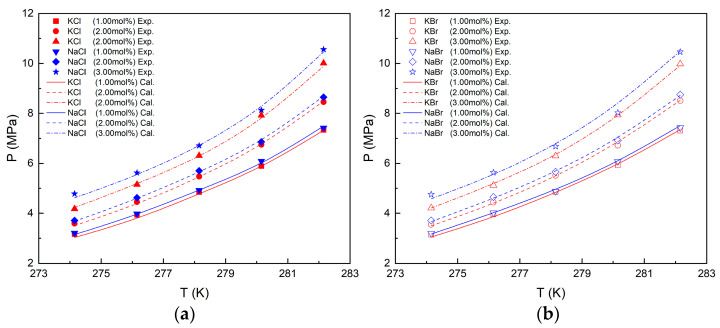
Experimental and predicted results of VL_W_H equilibrium conditions in different solutions. (**a**) Experimental and predicted results in KCl and NaCl solutions. (**b**) Experimental and predicted results in KBr and NaBr solutions.

The VL_W_H equilibrium conditions of CH_4_ hydrate in various inorganic salt systems were predicted using the Chen–Guo model and N–NRTL–NRF activity model. The detailed derivation of the models is shown in Appendix B. The VL_W_H equilibrium condition prediction curve and experimental data are presented in Figure 3. It is evident from the results that the experimental data align well with the predicted curves when inorganic salts are present. The average absolute relative deviation (*AARD*) calculated by Equation (1) describes the overall accuracy of the model’s predictions, as shown in Table 3. For CH_4_ hydrate, the *AARD* for KCl, NaCl, KBr, and NaBr solution systems is less than 1.24%, 1.08%, 1.18%, and 1.21%, respectively, indicating a high level of overall prediction accuracy for the model.

(1) $$AARD\left(\%\right)=\sum_{i=1}^{n}\left|\frac{P_{exp,i}-P_{pre,i}}{P_{exp,i}}\right|/n\times 100\%$$

where $P_{exp,i}$ and $P_{pre,i}$ are the VL_W_H equilibrium pressure of experimental and prediction, respectively. It is worth noting that, because the calculation of model parameters such as the Langmuir constant ($C_{i}$) adopts the method of empirical formula fitting as shown in Equation (A9), this method has certain limitations. The model should not be used beyond the data range investigated in this work. It is found that the error range of the model is within 4.42% in the pressure range of 2.84 MPa–10.56 MPa.

## 3. Materials and Methods

### 3.1. Experimental

#### 3.1.1. Materials

The properties and details of material used in this study are listed in Table 4. Experimental solutions were prepared using deionized water. The weights of materials were weighted using an electronic balance model LQ-A5003 with an accuracy of ±0.001 g.

#### 3.1.2. Experimental Apparatus

**Figure 4:**
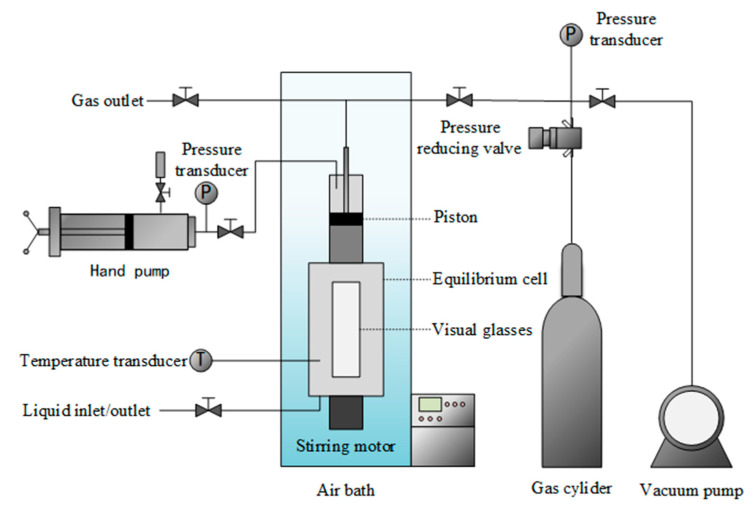
Schematic diagram of the experimental setup.

The experimental setup delineated in Figure 4 comprises a high-pressure equilibrium cell featuring a magnetic stirrer. The equilibrium cell, boasting a volumetric capacity of 200 mL, operates at a maximum working pressure of 30.0 MPa. The equilibrium cell crafted predominantly from sapphire serves as the locus for both hydrate formation and dissociation processes. A servo motor is used to control the rotational speed of the magnetic stirring to optimize mass transfer efficiency. Additionally, a manually controlled piston, modulated via a hand pump, regulates volume alterations within the equilibrium cell, thereby exerting control over internal gas pressure levels. Pressure measurements are facilitated by a Senex DG2111-C-20 pressure transmitter, boasting a maximum error margin of ±0.02 MPa. Temperature regulation within the equilibrium cell is achieved through the utilization of a cooling air bath in the range of 243.15 to 333.15 K. Temperature monitoring is facilitated by an Omega Pt100 platinum resistance thermometer with accuracy of ±0.01 K.

#### 3.1.3. Experimental Procedures

The “isothermal pressure search method” was used to measure the equilibrium conditions of VL_W_H [26]. The first step was to wash the equilibrium cells three times with the prepared solution. After washing the cells, 40 mL of the prepared solution was inhaled, and then the whole experiment was filled with CH_4_ and pumped three times to replace residual air. After this, an amount of CH_4_ was injected up to the pressure of the experiment. The temperature of the gas and solution in the equilibrium cell was then cooled to the target temperature by turning the air bath. Stirring was switched on during the experiment to maintain a constant stirring rate. Turning the hand pump increased the gas pressure in the equilibrium cell and hydrates formed rapidly. Then, the hand pump was rapidly turned to reduce the gas pressure until the hydrate was completely dissociated. The gas pressure was then slowly increased at a gradient of 0.02 MPa until the appearance of trace particles of hydrate in the equilibrium cell. At this point, the hand pump stopped operating. If the gas pressure and hydrate particles remain constant for 4 h, the temperature and pressure at this point are the VL_W_H equilibrium conditions. By repetition of the above steps, the VL_W_H equilibrium conditions can be measured for different additive solutions. The measurements should be repeated three times for each of the experimental conditions and the median value taken.

## 4. Conclusions

In order to investigate the influence of four inorganic salts on the thermodynamic conditions of methane hydrate formation, the VL_W_H equilibrium conditions of CH_4_ hydrate in KCl, NaCl, KBr and NaBr solutions at different concentrations were determined in the temperature range of 274.15 K–282.15 K. The results demonstrate that the four inorganic salts can significantly reduce the phase equilibrium pressure of CH_4_ hydrate and are effective thermodynamic inhibitors of methane hydrate. Furthermore, it was found that Na^+^ in cations exhibited stronger thermodynamic inhibition than K^+^. However, there was no discernible difference between Cl^−^ and Br^−^ in inhibiting methane hydrate. Additionally, the $∆H_{diss}$ of CH_4_ hydrate in the four inorganic salt systems and deionized water systems was found to be essentially identical. This indicates that the inorganic salts do not participate in the construction of the crystal structure of the hydrate. A model based on the Chen–Guo model and the N–NRTL–NRF activity model was employed to accurately predict the equilibrium conditions of CH_4_ hydrate in the presence of an electrolyte solution. The AARD values between the model prediction and the experiment were 1.24%, 1.08%, 1.18%, and 1.21%, respectively. The model demonstrated superior performance. This study offers insights into the mechanism and model prediction of inorganic salt inhibition of hydrate.

## Figures and Tables

**Table 1:** VL_W_H equilibrium conditions of CH_4_ in inorganic salt systems *.

System	1.00 mol%		2.00 %		3.00%	
	*T*/K	*P*/MPa	*T*/K	*P*/MPa	*T*/K	*P*/MPa
KCl	274.15	3.17	274.15	3.59	274.15	4.18
	276.15	3.94	276.15	4.45	276.15	5.15
	278.15	4.85	278.15	5.47	278.15	6.31
	280.15	5.89	280.15	6.75	280.15	7.92
	282.15	7.34	282.15	8.46	282.15	10.01
NaCl	274.15	3.21	274.15	3.71	274.15	4.58
	276.15	3.98	276.15	4.61	276.15	5.42
	278.15	4.92	278.15	5.68	278.15	6.71
	280.15	6.09	280.15	6.96	280.15	8.44
	282.15	7.52	282.15	8.82	282.15	10.71
KBr	274.15	3.15	274.15	3.55	274.15	4.21
	276.15	3.96	276.15	4.44	276.15	5.12
	278.15	4.86	278.15	5.51	278.15	6.3
	280.15	5.92	280.15	6.72	280.15	7.94
	282.15	7.31	282.15	8.51	282.15	9.98
NaBr	274.15	3.25	274.15	3.68	274.15	4.61
	276.15	4.02	276.15	4.58	276.15	5.38
	278.15	4.88	278.15	5.72	278.15	6.73
	280.15	6.12	280.15	6.98	280.15	8.42
	282.15	7.54	282.15	8.77	282.15	10.72

* u(*P*) = ±0.02 MPa, u(*T*) = ±0.01 K, u(*c*) = ±0.01 mol%.

**Table 2:** $∆H_{diss}$ of CH_4_ hydrate in inorganic salts systems of different concentrations *.

System	*c*/mol%	*T*/K	*P*/MPa	*z*	∆*H_diss_*/kJ·mol^−1^
Deionized water	/	273.15–282.15	2.84~6.46	0.9309~0.8697	−59.42
KCl	1.00	273.15–282.15	3.17~7.34	0.9231~0.8553	−59.80
	2.00	273.15–282.15	3.59~8.46	0.9133~0.8387	−60.40
	3.00	273.15–282.15	4.18~10.01	0.8999~0.8195	−62.21
NaCl	1.00	273.15–282.15	3.21~7.42	0.9222~0.8330	−59.30
	2.00	273.15–282.15	3.71~8.65	0.9106~0.8121	−57.84
	3.00	273.15–282.15	4.78~10.56	0.8865~0.7864	−58.52
KBr	1.00	273.15–282.15	3.15~7.31	0.9236~0.8350	−58.98
	2.00	273.15–282.15	3.55~8.51	0.9143~0.8143	−60.11
	3.00	273.15–282.15	4.21~9.98	0.8992~0.7932	−58.89
NaBr	1.00	273.15–282.15	3.20~7.43	0.9224~0.8328	−59.22
	2.00	273.15–282.15	3.71~8.75	0.9106~0.8105	−58.48
	3.00	273.15–282.15	4.75~10.46	0.8871~0.7875	−58.03

* u(*P)* = ± 0.02 MPa, u(*T*) = ±0.01 K, u($∆H_{diss}$) = ±0.01 kJ·mol^−1^.

**Table 3:** Predicted results of VL_W_H equilibrium conditions for CH_4_ hydrate *.

System	*c*/mol%	*N*	*AARD*/%
KCl	1.00	5	1.24
	2.00	5	
	3.00	5	
NaCl	1.00	5	1.08
	2.00	5	
	3.00	5	
KBr	1.00	5	1.18
	2.00	5	
	3.00	5	
NaBr	1.00	5	1.21
	2.00	5	
	3.00	5	

* *N* is the amount of experimental data.

**Table 4:** Details of the experimental materials used in this study *.

Chemical	Chemical Structure	Purity	Supplier	CAS
Potassium chloride	KCl	99.99%	Shanghai Aladdin Biochemical Technology Co., Ltd. (Shanghai, China)	7447-40-7
Sodium chloride	NaCl	99.99%	Shanghai Aladdin Biochemical Technology Co., Ltd. (Shanghai, China)	7647-14-5
Potassium bromide	KBr	99.99%	Shanghai Aladdin Biochemical Technology Co., Ltd. (Shanghai, China)	7758-02-3
Sodium bromide	NaBr	99.99%	Shanghai Aladdin Biochemical Technology Co., Ltd. (Shanghai, China)	7647-15-6
Deionized water	H_2_O	18.0MΩ·cm	Smart-Q15 ultrapure water machine (Suzhou, China)	732-18-5

* All chemicals were used without further purification.

## Data Availability

The data presented in this study are available upon request from the corresponding author.

## Appendix A

In order to investigate the thermodynamic properties of CH_4_ hydrates in the presence of inorganic salts, it is necessary to calculate the hydrate dissociation enthalpies ($∆H_{diss}$) of hydrate based on the VL_W_H equilibrium data using the Clausius—Clapeyron equation, which is a widely used equation in phase equilibrium studies. The equation for calculation is as follows [26,27]:

(A1) $$\frac{dlnP}{d\frac{1}{T}}=-\frac{∆H_{diss}}{zR}$$

where *T* and *P* denote equilibrium temperature and pressure, respectively. *z* represents the compressibility factor of the methane gas at experimental conditions. *R* is the universal gas constant, $J\cdot {mol}^{-1}\cdot K^{-1}$. The approach is predicated on thermodynamic relationships to inform the development of evaluation criteria. Thermodynamic consistency assessment includes the linear consistency, dissociation enthalpy consistency and the water activity consistency of the VL_W_H equilibrium data.

The linear consistency was quantified by measuring the concordance between the experimental data and the linear regression equation, as represented by Equation (A2) which is obtained by integrating Equation (A1). This was achieved by utilising the statistical goodness of fit (*R*^2^) to characterize the degree of closeness between the experimental data and the fitted regression equation, as calculated by Equation (A3).

(A2) $$\ln\left(P\right)=\frac{A}{T}-B$$

(A3) $$R^{2}=1-\frac{\sum {\left(y_{i}-{\hat{y}}_{i}\right)}^{2}}{{\sum \left(y_{i}-{\bar{y}}_{i}\right)}^{2}}$$

where *A* and *B* are obtained by regression of VL_W_H equilibrium temperature and pressure. ln⁡(P) is a linear function of $\frac{1}{T}$ after integration by Equation (A1). The parameter *A* is a constant corresponding to $∆H_{diss}$ and z. Values of A will be used for assessing the thermodynamic consistency of the $∆H_{diss}$, as shown in Equation (A4). The parameter *B* is the constant corresponding to the intercept of Equation (A2), i.e., the integral constant after the integration of Equation (1). yi, ŷi and $\bar{y}$ denote the experimental results, calculated results by the fitting equation, and the average of the experimental results, respectively.

The dissociation enthalpy consistency was evaluated by examining the proportional differences between $A_{THI}$ and $A_{W}$, as illustrated by Equation (A4). $A_{THI}$ and $A_{W}$ represent the gradients of the linear fitting regression equations for the salt-containing system and the deionized water system, respectively. If the values of ($A_{THI}$ − $A_{W}$)/$A_{W}$ are all found to be within a range of ±5.0%, it suggests that there exists a high level of thermodynamic concordance when considering the constraint associated with the value of $∆H_{diss}$.

(A4) $$A=-\frac{{∆H}_{diss}}{zR}$$

The water activity consistency was evaluated, with a specific focus on the influence of the THIs’ type and concentration on the outcome. The hydrate suppression temperature ($∆T=T_{0}-T$) in inorganic salts systems is expressed as Equation (A5). If the relative standard deviations (RSD) of $\frac{∆T}{T_{0}T}$, which are used to quantify the thermodynamic consistency of the $a_{w}$, are all within 10% in inorganic salt systems, it suggests that there exists a high level of water activity consistency in term of the $a_{w}$ constraint [28].

(A5) $$\frac{∆T}{T_{0}T}=-\frac{nR}{{∆H}_{diss}}\ln a_{w}$$

where *T* is the VL_W_H equilibrium temperature for the THI systems, and $T_{0}$ is the VL_W_H equilibrium temperature for the deionized water systems. n is the hydration number.

## Appendix B

### Appendix B.1. The Chen–Guo Thermodynamic Model

The Chen–Guo model was based on the assumption of a double process hydrate initiation mechanism [29]. The double process hydrate formation mechanism includes quasi-chemical reaction and Langmuir adsorption process [30]. The quasi-chemical reaction and adsorption process can be described by the Equations (A6) and (A7), respectively [31].

(A6) $$H_{2}O+{\lambda_{2}CH}_{4}\to H_{2}O\cdot {{CH}_{4}}_{\lambda_{2}}$$

(A7) $$H_{2}O\cdot {{CH}_{4}}_{\lambda_{2}}+\lambda_{1}\cdot \theta_{{CH}_{4}}\cdot {CH}_{4}\to H_{2}O\cdot {{CH}_{4}}_{\lambda_{2}}\cdot {{CH}_{4}}_{\lambda_{1}\cdot \theta_{{CH}_{4}}}$$

where $\lambda_{1}=1/23$ and $\lambda_{2}=3/23$ for structure I. θ is the fraction of the gas molecules in the cavities, which is calculated by Equations (A8) and (A9).

(A8) $$\theta_{i}=\frac{C_{i}f^{gas}}{1+C_{i}f^{gas}}$$

(A9) $$C_{i}=Xexp(\frac{Y}{T-Z})$$

where $C_{i}$ is the Langmuir constant for CH_4_. The $X=2.3048\times 10^{-11}{Pa}^{-1}$, Y = 2752.29 K and Z = 23.01 K for CH_4_ hydrate, respectively. T is the temperature of the VL_W_H equilibrium condition. According to the phase equilibrium criterion, the fugacity of gas component in the gas phase ($f_{i}^{g}$) should be equal to the fugacity in the hydrate phase ($f_{i}^{H}$). $f_{i}^{g}$ is calculated by the P-T EOS. $f_{i}^{H}$ is calculated by the Equation (A10).

(A10) $$f_{i}^{H}=f^{0}{\left(1-\theta \right)}^{\alpha }$$

(A11) $$f^{0}=f_{T}^{0}exp(\frac{\beta P}{T})a_{w}^{-1/\lambda_{2}}$$

(A12) $$f_{T}^{0}=Aexp(\frac{B}{T-C})$$

where $f^{0}$ is a function of *T*, *P*, $a_{w}$ and hydrate structure. $f_{T}^{0}$ is a function of temperature and can be related by the Antoine equation. For CH_4_ hydrate, α=1/3. $\beta =4.242\times 10^{-6}K{\cdot Pa}^{-1}$. *A* = $1.5844\times 10^{18}\;Pa$, *B* = −6591.43 K and *C* = 27.04 K, respectively [32].

### Appendix B.2. Water Activity ($a_{w}$) Model

$a_{w}$ is calculated by the N–NRTL–NRF activity model in this study. The contributions of dissolved gas and electrolytes are considered to the $a_{w}$ as the Equation (A13) [33]:

(A13) $$a_{w}=a_{w,g}a_{w,el}$$

where $a_{w,g}$ and $a_{w,el}$ are the contributions of dissolved gas and electrolytes to $a_{w}$, respectively. The dissolved CH_4_ is very small, so that the $a_{w,g}$ takes the value of 1. $a_{w,el}$ is calculated by the Pitzer–Debye–Hückel equation as in Equations (A14)–(A16) [34]:

(A14) $$a_{w,el}=x_{w}\gamma_{w}$$

(A15) $$x_{w}=\frac{x_{w,0}}{x_{w,0}+vx_{el,0}}$$

(A16) $$x_{el}=1-x_{w}$$

where $x_{w}$ and $x_{el}$ denote the molar concentrations of water and ions in solution, respectively. $x_{w,0}$ and $x_{el,0}$ denote the molar concentrations of water and electrolytes in solution. v is the stoichiometric number of ions. $\gamma_{w}$ is the activity coefficient of water, which is calculated as Equation (A17).

(A17) $$\ln\gamma_{w}=\ln\gamma^{SR}+\ln\gamma^{LR}$$

where $\gamma^{SR}$ is the short-range correction term, which is calculated by Equations (A18)–(A24) [33].

(A18) $$\ln\gamma^{SR}=x_{el}^{2}(\tau_{el,w}\Gamma_{el,w}^{2}+\frac{\tau_{w,el}\Gamma_{el,w}^{2}}{G_{w,el}}-\tau_{el,w}-\tau_{w,el})$$

(A19) $$\tau_{w,el}=\frac{g_{w,el}-g_{el,el}}{RT}$$

(A20) $$\tau_{el,w}=\frac{g_{el,w}-g_{w,w}}{RT}$$

(A21) $$G_{w,el}=exp(-\alpha \tau_{w,el})$$

(A22) $$G_{el,w}=exp(-\alpha \tau_{el,w})$$

(A23) $$\Gamma_{w,el}=\frac{x_{w}G_{w,el}}{x_{w}G_{w,el}+x_{el}}$$

(A24) $$\Gamma_{el,w}=\frac{x_{el}G_{el,w}}{x_{el}G_{el,w}+x_{w}}$$

where $g_{i,j}$ and $\tau_{i,j}$ are the interaction parameters and the energy parameter. $G_{i,j}$ and $\Gamma_{i,j}$ are the Boltzmann factor and the nonrandom factors. α=0.3. $\gamma^{LR}$ is the long-range correction term, which is calculated by Equations (A25) and (A26) [33].

(A25) $$\ln\gamma^{LR}=\frac{x_{el}}{x_{w}}A_{\phi }{\left(\frac{1000}{M_{w}}\right)}^{\frac{1}{2}}(\frac{\left|z_{a}z_{c}\right|I^{\frac{1}{2}}-2I^{3/2}}{1+\rho I^{1/2}})$$

(A26) $$I=\frac{1}{2}(x_{a}z_{a}^{2}+x_{c}z_{c}^{2})$$

where $x_{a}$, $x_{c}$ and $z_{a}$, $z_{c}$ are the molar concentrations and charge number of cations and anions, respectively. I represents the ionic strength. The Debye–Hückel constant $A_{\phi }$ is equal to 0.390947 and the proximity ρ is equal to 14.90, respectively [35].
